# Quantitative Assessment of the Restoration of Original Anatomy after 3D Virtual Reduction of Long Bone Fractures

**DOI:** 10.3390/diagnostics12061372

**Published:** 2022-06-02

**Authors:** Moo-Sub Kim, Do-Kun Yoon, Seung-Han Shin, Bo-Young Choe, Jong-Won Rhie, Yang-Guk Chung, Tae Suk Suh

**Affiliations:** 1 Department of Biomedical Engineering and Research Institute of Biomedical Engineering, College of Medicine, Catholic University of Korea, Seoul 06591, Korea; mskim0726@naver.com (M.-S.K.); bychoe@catholic.ac.kr (B.-Y.C.); 2 Industrial R&D Center, KAVILAB Co., Ltd., Seoul 06693, Korea; louis_youn@kavilab.ai; 3 Department of Orthopedic Surgery, College of Medicine, Catholic University of Korea, Seoul 06591, Korea; tumorshin@gmail.com; 4 Department of Plastic Surgery, College of Medicine, Catholic University of Korea, Seoul 06591, Korea; rhie@catholic.ac.kr

**Keywords:** virtual reduction, 3D image, surgical plan, 3D image accuracy, radial fractures

## Abstract

*Background:* The purpose of this study was to demonstrate the usefulness of 3D image-based virtual reduction by validating the evaluation criteria according to guidelines suggested by the AO Surgery Reference. *Methods:* For this experiment, 19 intact radial ORTHObones (ORTHObones radius, 3B Scientific, Germany, Hamburg) without any fractures were prepared. All ORTHObones with six cortical marking holes (three points on the distal part and three points on the proximal part) were scanned using a CT scanner twice (before/after intentional fracture of the ORTHObone). After the virtual reduction of all 19 ORTHObones, accuracy evaluations using the four criteria (length variation, apposition variation, alignment variation, Rotation Variation) suggested in the AO Surgery Reference were performed. *Results:* The mean (M) length variation was 0.42 mm, with 0.01 mm standard deviation (SD). The M apposition variation was 0.48 mm, with 0.40 mm SD. The M AP angulation variation (for alignment variation) was 3.24°, with 2.95° SD. The M lateral angulation variation (for alignment variation) was 0.09°, with 0.13° SD. The M angle of axial rotation was 1.27° with SD: 1.19°. *Conclusions:* The method of accuracy evaluation used in this study can be helpful in establishing a reliable plan.

## 1. Introduction

Purpose of fracture treatment is to achieve the union of the bone fragments [1,2,3,4,5,6,7,8,9]. Moreover, it is to restore the function of the musculoskeletal system and prevent future complications associated with the failure of restoration of the normal anatomy [10,11,12,13]. The reduction of a fracture is the process of reconstructing the fractured bone by relocating the pieces of the fractured bones in their original positions [14,15,16,17]. Because the prognosis of the fracture can be concluded by the performance of the reduction, reduction is the one of most significant steps in the surgery [18,19]. Although there have been many recent studies regarding reductions using three-dimensional (3D) images or 3D printing, most of the studies have reported results through tactile modeling or mirroring techniques [20]. Moreover, the authors in those studies concluded good performance using their own measures of success without quantified assessments [1,21,22].

Conventional surgical simulation means brainstorming to establish surgical strategy, usually using 3D computed tomography (CT) images of broken bones, rather than 3D-image-based reduction of the fractures [23,24]. Because most of the current viewer systems are based on the picture archiving and communication system (PACS) used in most medical institutions, the function of editing or modeling 3D objects is not available and pre-surgical simulation with the virtual reduction of bone fractures (hereafter referred to as “3D-image-based reduction”) was not originally possible [25,26,27,28,29,30]. Thus, the accuracy and reliability of 3D image-based virtual reduction using quantitative assessment also could not be determined using that software. If 3D-image-based virtual reduction can aid in the restoration of the pre-injury anatomy of fractured bones, it will be able to provide a model for 3D-printed implant fabrication and can be used as a highly reliable pre-surgical simulation and fracture management tool. A more accurate surgical approach can be realized with greatly reduced operation times, minimizing the associated surgical complications [31,32,33,34,35,36,37,38,39]. However, to be helpful to the clinical field, the verification of the accuracy of 3D-image-based virtual reduction should be determined. Although there are several analysis methods for 3D objects, we found a suitable analysis method through the quantitative approach with clinical guidelines for 3D anatomical bone structures after reduction [40].

3D image-based virtual reduction can be helpful because orthopedic surgeons can confirm the eventual shape of the reduced bone before the actual operation. That result can be derived using the 3D modeling tool, or it can be automatically generated by dedicated software such as KAVIPLAN (Ver 1.0, KAVILAB Co., Ltd., Seoul, Korea). However, to refer the results of the 3D image-based virtual reduction to the actual surgery, the accuracy of the results should be presented as the numerical values to confirm the reliability. The actual reduction process is manually being progressed with the only naked eye, like doing a 3D puzzle. And there can be some bone fragments which are occluded by the soft-tissue such as blood vessels, nerves, etc. Moreover, surgeons normally can see only the restricted part by an incision. For these reasons, it is not easy to progress the actual reduction when the fracture case includes a high complexity level or the fracture is generated on the difficult region. However, if the surgeon can perform the difficult reduction including even the occluded fragments by the soft-tissue with seeing the 3D image for the reduced bone, the 3D puzzle can be done with seeing the feature of the true structure. The results of this study and its trend can provide an opportunity to get insight for effective surgical strategy as follows. If orthopedic surgeons can have faith for the final shape of virtually reduced bone, it will be clearly helpful to select the kind of fixation device, and to expect its location. Although the indefinite final shape of virtually reduced bone can be provided, orthopedic surgeons are not easy to have confidence to correctly decide which one is the best device for the surgery without the correct shape. In that case, they have to depend on the final shape by the actual reduction rather than use of virtual reduction during the operation. Moreover, many times of change for the kind of the device and its shape to fit the shape on the bone will be essential. These kinds of concerns are caused by the indefinite final shape for the reduced bone by the expectation. The pre-operative surgical plan including the virtual reduction without the accuracy will make the meaning of planning weak. The purpose of this study was to demonstrate the usefulness of 3D image-based virtual reduction by validating the evaluation criteria according to guidelines suggested by the AO Surgery Reference. All of the results in this study were based on numerical values measured in a 3D coordinate system with quantified assessments.

## 2. Materials and Methods

### 2.1. Material Preparation and CT Scanning

**Figure 1:**
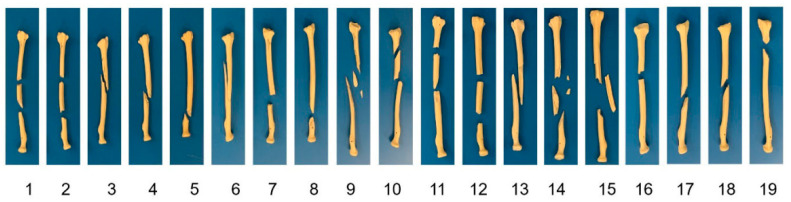
Experimental fracture models for 19 ORTHObone tag numbers. The simple fractures (11 cases) involved only two fragments, while the comminuted fractures (8 cases) involved three or more fragments. Each classification included both transverse fractures (4 cases) and spiral fractures (15 cases).

For this experiment, 19 intact radial ORTHObones (ORTHObones radius, 3B Scientific, Hamburg, Germany) without any fractures were prepared. Each ORTHObone was tagged with a number from 1 to 19. The average size was 25 cm (H), 7 cm (W), and 3 cm (T), to reflect a typical adult’s radius bone. Each ORTHObone had small size and shape variations, and both the rigidity and density of the ORTHObones were similar to those of human bones. Firstly, ORTHObones with six cortical marking holes (three points on the distal part and three points on the proximal part) were scanned using a CT scanner (120 kVp at 100 mAs; Siemens, SOMATOM Definitions AS+, Munich, Germany). Moreover, the three center points of these marking holes satisfied a minimal condition to make a plane for generating a normal vector, which is the signature of 3D angulation. CT images were acquired with a 0.75-mm slice thickness according to the digital imaging and communications in medicine (DICOM) format with a 512 × 512 pixel matrix. All DICOM images segmented for only part of the bone were reconstructed as 3D images using dedicated MIMICS software (Ver 21.0, Materialise, Leuven, Belgium). The next step was scanning after the intentional fracture of the ORTHObone. Many fracture patterns are possible depending upon conditions such as stress and the force vector. Obviously, the trend of actual fracture is too various to replicate to all ORTHObones in this study. Instead, the regions of fracture were classified as proximal fracture, mid-shaft fracture and distal fracture. And if the number of bone fragments is over two pieces, that case is classified as the comminuted fracture. Moreover, the pattern of fracture is classified as the spiral pattern, oblique pattern, and transverse pattern. In order to reflect all classifications as possible, the experimenter who is one of the authors intentionally gave forces as various methods to each ORTHObone, such as compressing, bending, twisting, and universal crushing stress. As a result, the various fractures for the ORTHObones were accomplished according to our intention. However, we intentionally used simplified classifications, namely a simple fracture (2 pieces; a total of 11 cases) and a comminuted fracture (more than 3 pieces; a total of 8 cases) [41]. When an external force is applied to the ORTHObone in a relatively perpendicular direction, the probability of a transverse fracture occurring is high. Otherwise, when the external force is applied with twisting, the probability of a spiral fracture occurring is high. Each fracture classification included both transverse (4 cases) and spiral patterns (15 cases), as shown in Figure 1.

### 2.2. Virtual Reduction and Accuracy Evaluation

**Figure 2:**
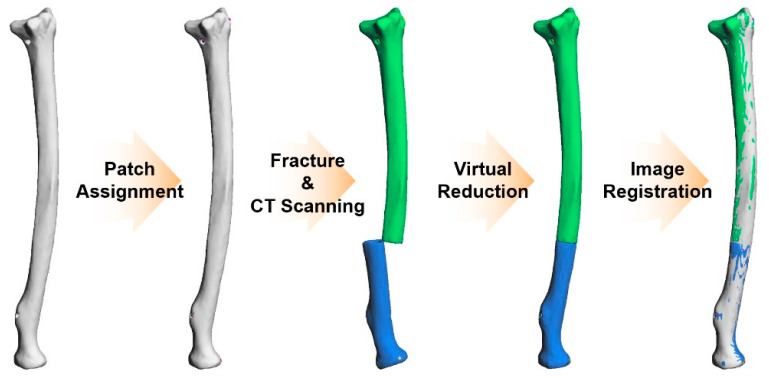
Scheme of the workflow for virtual reduction and image registration between the virtually reduced object and the original object. (1) CT scanning of the original bone, (2) assignment of patches at the cortical marking holes, (3) fracture of the ORTHObone, (4) CT scanning of the fractured bone, (5) virtual reduction, and (6) image registration.

The reduction of the fractured bone is a relocation of each bone fragment at the appropriate position during surgery. Likewise, a virtual reduction is a work process for relocating 3D bone fragment objects using a 3D modeling tool or a 3D space-based editing tool. Mostly, a virtual reduction is used for establishing a reduction plan for trauma surgery. Virtual reductions can be more effective in fractured bone models with high complexity. In this study, the virtual reduction for all 19 ORTHObones was performed manually using the 3D modeling tool Metasequoia 4 (Tetraface, Ver 4.7.0, Tokyo, Japan). The 3D reconstructed bone images from MIMICS were converted to the stereolithography (STL) format and then imported into Metasequoia 4. Because the 3D object of each bone fragment was already separated from MIMICS, the virtual reduction was conducted by intuitively operating each 3D object, such as moving and rotating. When a virtual reduction is performed using the 3D modeling tool, because there is no tactile sensation like in an actual reduction, virtual reductions depend only on the shape of the ultimate feature. For this reason, an accurate evaluation of the virtual reduction is essential. After the virtual reduction of all 19 ORTHObones, accuracy evaluations using the four criteria suggested in the AO Surgery Reference were performed. The accuracy evaluation compared the image registration between the 3D object for the original bone and the 3D object after the virtual reduction. To adjust the location of the virtually reduced objects to the location of the original bone, the three cortical marking holes on the distal part of the radius were used. When the three cortical marking holes on the distal part were matched between the virtually reduced object and the original object, the dislocation of the other objects was easily revealed by the variation in position (such as the length and angle) of the three remaining marking holes on the proximal part. Figure 2 shows a process of preparation for evaluating the accuracy of virtual reduction. 

**Figure 3:**
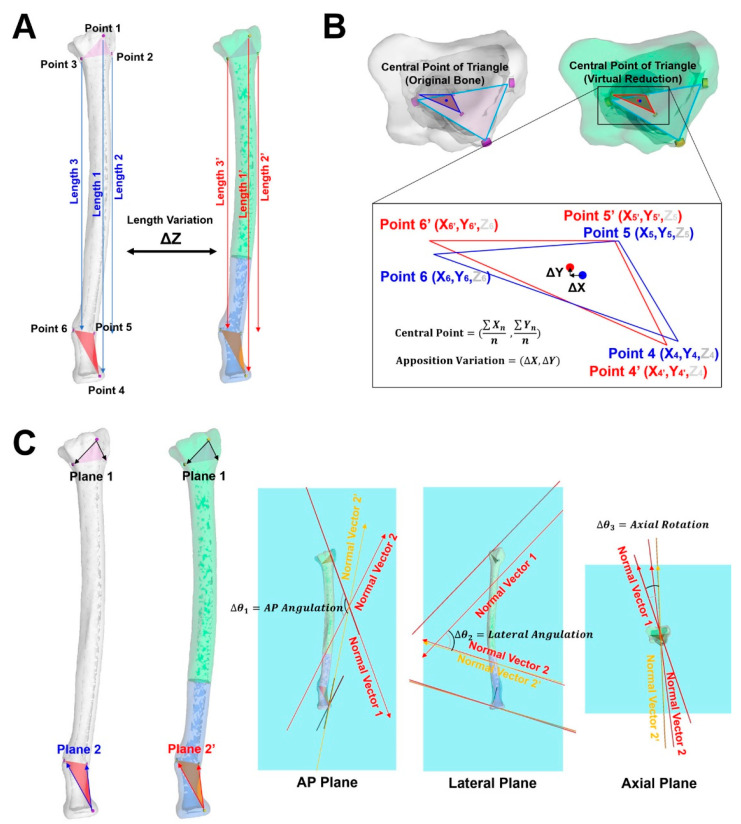
3D-based method of accuracy evaluation for virtual reduction. (**A**) There were three cortical marking holes on the distal part and three points on the proximal part. The three original lengths only in the Z-axis were measured as Length 1 (between Point 1 and Point 4), Length 2 (between Point 2 and Point 5) and Length 3 (between Point 3 and Point 6). The length variation was calculated using the change in length (Length 1′, Length 2′, and Length 3′). (**B**) When the 2D sky blue color triangles for both the original object and the virtually reduced object were fixed at the same position, the location of the deep 2D blue triangle belonging to the original object was converted to the location of the 2D red triangle in the virtually reduced object. The variation of apposition was calculated by measuring the variation of the X and Y coordinates from the central point of the triangles (from deep blue to red) (**C**) The three marking holes on the distal part generated Normal Vector 1 via Plane 1, and the other three marking holes on the proximal part generated Normal Vector 2 via Plane 2 (from original object), and Normal Vector 2′ via Plane 2′ (from the virtually reduced object). AP angulation and Lateral angulation indicate the difference in the angle between Normal Vector 2 and Normal Vector 2′ on the AP plane and the lateral plane, respectively. The angle difference in the rotation was measured by the variation in the angle between Normal Vector 2 and Normal Vector 2′ on the axial plane.

The first evaluation was performed by measuring the vertical length between the centers of the marking holes. Figure 3A shows the method used to measure the length between the centers of the marking holes and the variation. The distance between Points 1 and 4, 2 and 5, and 3 and 6 were measured. When the Z-axis of the 3D modeling tool agreed with the long axis of the long bone, the length in only the Z-axis direction was measured and the variation between the original length and the length after virtual reduction was calculated.

The second evaluation was a verification of the apposition, which can show the location variation of objects on the XY plane. Figure 3B shows the method of the accuracy evaluation of apposition in this study. When the viewpoint of the object in the 3D space was fixed according to the Z-axis, the sky-blue 2D triangle shown in Figure 3B composed of the centers of the three marking holes (Point 1, Point 2, and Point 3) was observed. Moreover, the deep blue triangle in Figure 3B (Point 4, Point 5, and Point 6) in the original object and the red triangle (Point 4′, Point 5′, and Point 6′) from the virtually reduced object were also observed. The central point of each triangle was used to calculate the variation in apposition. Because the apposition is based on the variation in the 2D coordinate, the Z-coordinate was excluded from the consideration of apposition. The apposition variation was calculated by the change in the central point from the blue point on the blue triangle to the red point on the red triangle after the virtual reduction.

Although the third and last evaluation corresponded to the measurements of alignment and rotation, respectively, both evaluations are performed at once using 3D angulation. Because 3D angulation includes three angles for the X-axis, Y-axis, and Z-axis, 2D angles can be measured when the 3D angulation vector is projected onto a 2D plane with one reference axis, which is included on that plane. For example, when there is a 3D angulation vector with X, Y, and Z coordinates, if the Z-coordinate is changed to 0, the angulation vector will be a 2D vector on the XY plane. To measure both the alignment and rotation of the bone by converting to 2D vectors, a 3D angulation vector induced by the three cortical marking holes was required. The bone objects on the left side of Figure 3C show the set of planes used to generate the 3D angulation vectors. Plane 1 was a plane composed of two vectors (Point 1 → Point 2 and Point 1 → Point 3). Normal Vector 1, which was a vertical vector with Plane 1, was set. Using the same principle, Normal Vector 2 from Plane 2 (Point 4 → Point 5 and Point 4 → Point 6) originated from the original object and Normal Vector 2′ (Point 4′ → Point 5′ and Point 4′ → Point 6′) from Plane 2′ belonged to the virtually reduced object. After the projection of the 3D angulation vectors (Normal Vectors) on the 2D plane, the intrinsic 2D angle between Normal Vector 1 and Normal Vector 2 was measured. The changed 2D angle was also measured using Normal Vector 1 and Normal Vector 2′. The variation in the 2D angle was calculated by the difference between the intrinsic 2D angle and the changed 2D angle on the same plane. In this study, we set the long axis of the long bone as the Z-axis. And because the bone objects on the left side of Figure 3C were set to the X-axis, the anteroposterior (AP) plane was set as the YZ plane, and the lateral and axial planes were set as the XZ and XY planes, respectively. Hence, we calculated the alignment variation from the variances in angulation in the AP plane and lateral plane. Moreover, the difference in the angle caused by rotation was calculated from the variance in angulation in the axial plane.

### 2.3. Statistical Analysis

For statistical analysis, the model of the paired *t*-test with an alpha of 0.05 was adopted using Statistics and Machine Learning Toolbox in MATLAB (R2021b, MathWorks, MA, USA). When we validated the standard normal distribution of the original object data using criteria that corresponded to both the mean and standard deviation (SD), the results for all raw data showed normality [42,43,44]. When the null hypothesis is rejected (h = 0), it means that two samples are not significantly different. However, if the null hypothesis is not ‘0′, there should be a clear difference between the samples.

## 3. Results

### 3.1. Ultimate Shape of the Virtual Reduction

**Figure 4:**
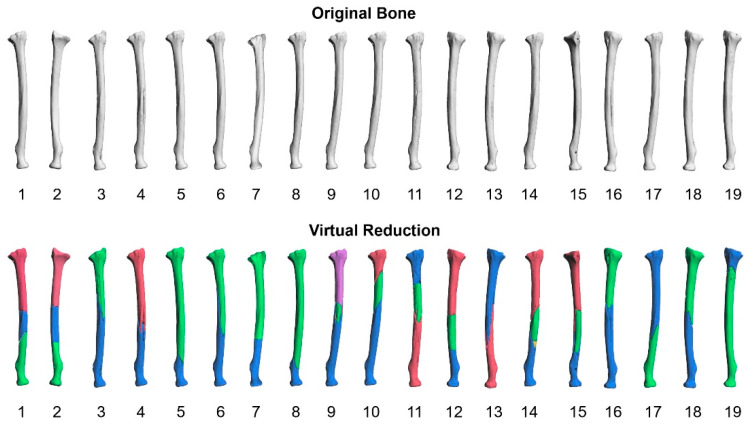
The original shapes of the 19 ORTHObones (top line) and the virtually reduced objects for all ORTHObones. The bone fragments in the virtually reduced object are represented by different colors (bottom line).

Figure 4 shows the ultimate shape of all virtually reduced objects for the fractured ORTHObones. The original shapes of the bones before the fractures are arranged at the top of Figure 4. The virtually reduced objects for the fractured bones are arranged at the bottom. Each bone fragment is represented in different colors such as blue, green, red, etc. It was difficult to visualize specific differences between the original shape and the shape of the virtually reduced object.

### 3.2. Length Variation

**Figure 5:**
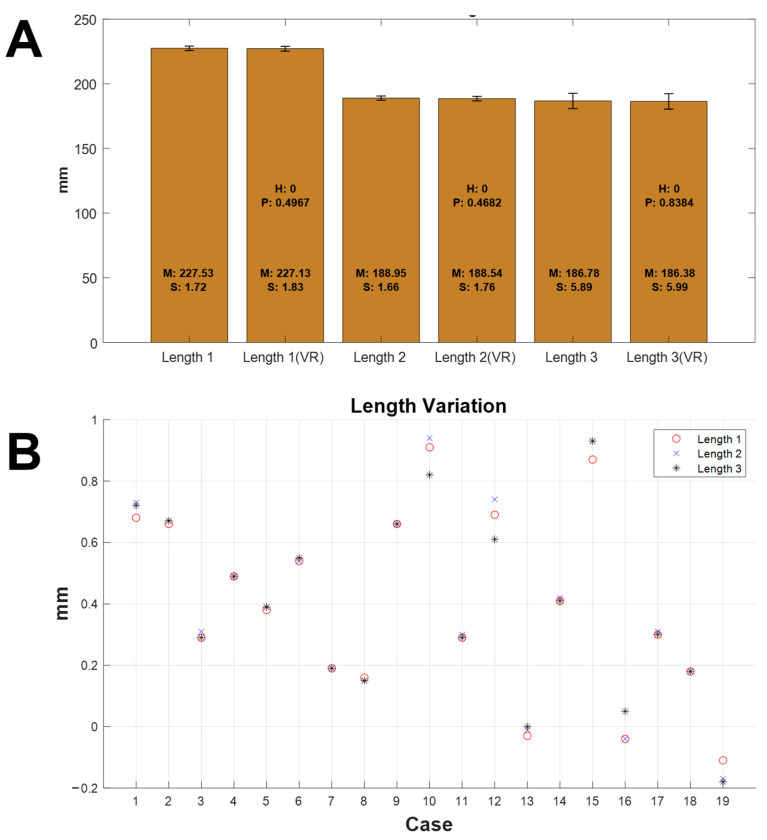
Results of the measured length and length variations between the original object and the virtually reduced object. (**A**) The measured lengths of specific distances (Length 1(′), Length 2(′), and Length3(′)) from the original object and the virtually reduced object. (**B**) The length variation in 19 ORTHObones before and after virtual reduction. VR: virtual reduction, M: mean, SD: standard deviation, H: null hypothesis, P: *p*-value.

Figure 5A shows the measured lengths of the original objects and the virtually reduced objects. Length 1, Length 2, and Length 3 are the distances between Points 1 and 4, Points 2 and 5, and Points 3 and 6, respectively. Both the mean and SD (S in the figure) shown on the bar in the graph were calculated using the results of the 19 cases. The means measured from the original object for Length 1, Length 2, and Length 3 were 227.53 mm (SD: 1.72 mm), 188.95 mm (SD: 1.66 mm), and 186.78 (5.89 mm). The means measured from the virtually reduced object for Length 1′, Length 2′, and Length 3′ were 227.13 mm (SD: 1.83 mm), 188.54 mm (SD: 1.76 mm), and 186.38 (5.99 mm), respectively. There were no statistical differences between the original object and the virtually reduced object in the three lengths analyzed by the paired *t*-test. The results for the individual variation in length according to each case are shown in Figure 5B. The mean length variation was 0.42 mm, with 0.01 mm SD. The maximum and minimum variations were 0.94 mm (Case 10) and 0.00 mm (Case 13), respectively. Length variation values on the graph below 0 indicated that the length of the virtually reduced object was shorter than that of the original object.

### 3.3. Apposition Variation

**Figure 6:**
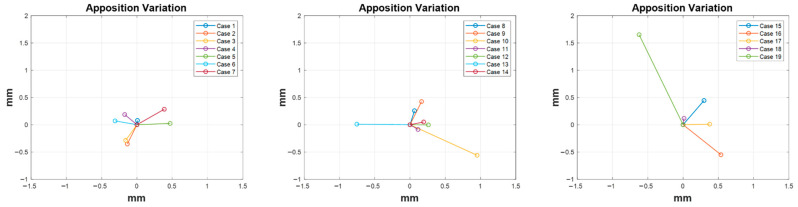
Apposition variation results after virtual reduction (the central position of the red triangle in Figure 3B) of the fractured bone. The central point of the deep blue triangle in Figure 3B was set at the central coordinate of the graph (X = 0, Y = 0). Left: Cases 1 to 7; middle: Cases 8 to 14; and right: Cases 15 to 19.

The apposition variation after virtual reduction was also measured (Figure 6). The graphs on the left, middle, and right show the results of apposition variation for Cases 1 to 7, Cases 8 to 14, and Cases 15 to 19, respectively. The central point of the deep blue triangle in Figure 3B was calibrated at X = 0, and Y = 0 on the graph and the apposition variation by the central point of the red triangle (in Figure 3B) is presented as a colored line. The mean apposition variation was 0.48 mm, with 0.40 mm SD. The maximum and minimum variations were 1.76 (Case 19) mm and 0.08 mm (Case 1), respectively. 

### 3.4. Alignment Variation

**Figure 7:**
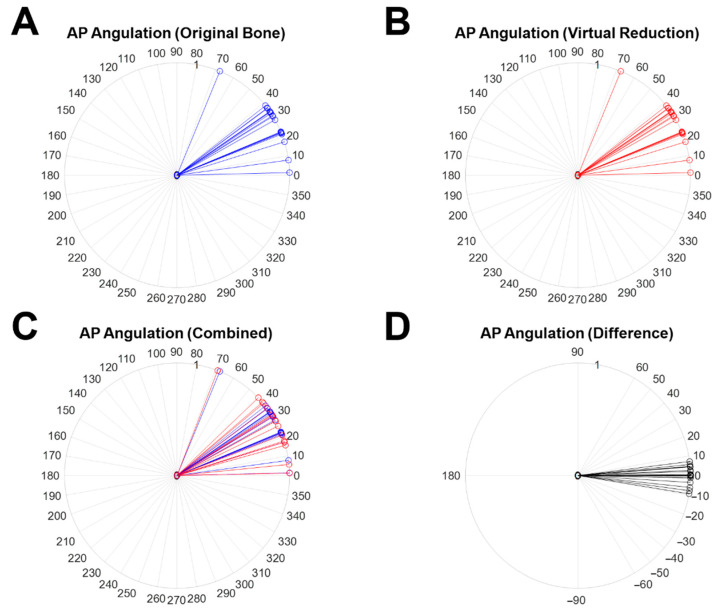
The measured 2D AP angulation on the YZ plane and the variation between the original object and the virtually reduced bone. (**A**) AP angulations measured from the angle between Normal Vector 1 and Normal Vector 2 (the original object), (**B**) AP angulations measured from the angle between Normal Vector 1 and Normal Vector 2′ (the virtually reduced object), (**C**) overlapped results for (**A**) with blue lines and (**B**) with red lines, and (**D**) the difference (black line) in AP angulation between (**A**) with blue lines and (**B**) with red lines.

The results of alignment variation based on angulation in the virtual reduction are shown in Figure 7. Each color line represents an individual AP angulation measured from the angle between Normal Vector 1 and Normal Vector 2(2′). The alignment variation was considered by the two kinds of angulations, AP angulation (Figure 7) and lateral angulation. Figure 7A,B present the AP angulation of the original object (blue line) and the virtually reduced object (red line), respectively. For intuitive comparison, both results overlapped on the same graph are presented in Figure 7C (blue line; AP angulation measured from the original object, red line; AP angulation measured from the virtually reduced object). The difference in AP angulation between the original object and the virtually reduced object is shown as a black line in Figure 7D. The mean AP angulation variation was 3.24°, with 2.95° SD. The maximum and minimum variations were 9.35° (Case 13) and 0.00° (Case 4), respectively. There was no statistical difference (h = 0) between the two samples with a *p*-value of 0.9744 analyzed by the paired *t*-test.

**Figure 8:**
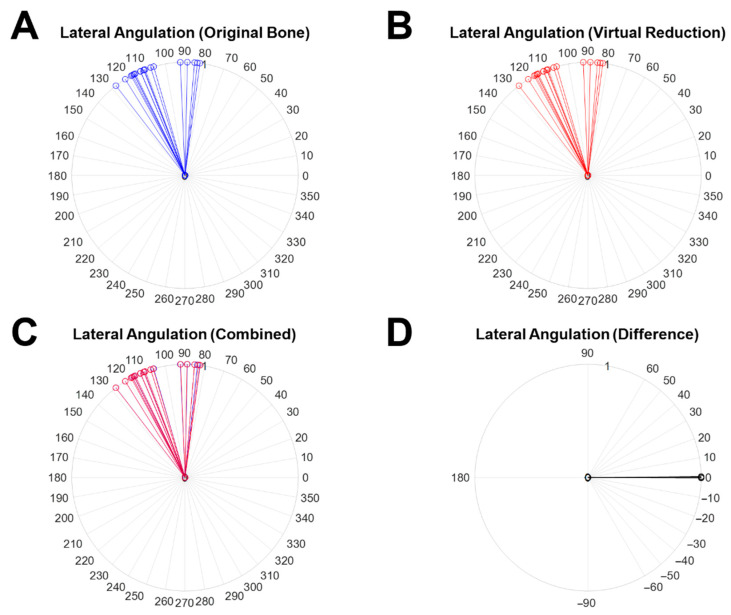
2D lateral angulation measured on the XZ plane and the variation between the original object and the virtually reduced bone. (**A**) The lateral angulations measured from the angle between Normal Vector 1 and Normal Vector 2 (the original object), (**B**) the lateral angulations measured from the angle between Normal Vector 1 and Normal Vector 2′ (the virtually reduced object), (**C**) overlapped results of (**A**) with blue lines and (**B**) with red lines, and (**D**) the difference (black line) in lateral angulation between (**A**) with blue lines and (**B**) with red lines.

Figure 8 shows the results of lateral angulation measured from the 2D angle on the XZ plane and the variation. Figure 8A, B present the lateral angulation of the original object (blue line) and the virtually reduced object (red line), respectively. The overlapping results of both cases (blue line; lateral angulation measured in the original object (red line; lateral angulation measured in the virtually reduced object) are shown in Figure 8C. The difference in the lateral angulations is shown as a black line in Figure 8D. The mean lateral angulation variation was 0.09° with SD: 0.13° The maximum and minimum variations were 0.54° (Case 19) and 0.00° (Case 4 and Case 18), respectively. There was no statistical difference (h = 0) between the two samples with a *p*-value of 0.9986 in the paired *t*-test.

### 3.5. Rotation Variation

**Figure 9:**
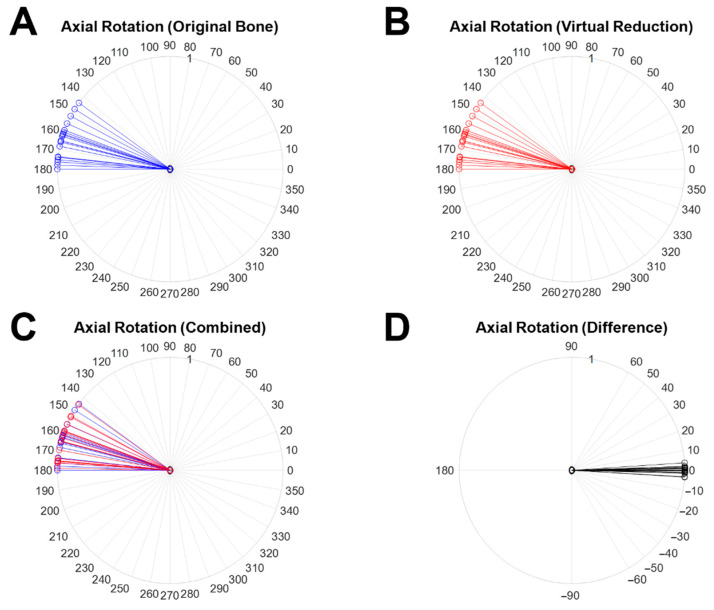
The measured 2D angle of axial rotation in the XY plane using 3D angulation and the variation between the original object and the virtually reduced bone. (**A**) The axial rotation measured from the angle between Normal Vector 1 and Normal Vector 2 (the original object), (**B**) the axial rotation measured from the angle between Normal Vector 1 and Normal Vector 2′ (the virtually reduced object), (**C**) the overlapped results for (**A**) with blue lines and (**B**) with red lines, and (**D**) the difference (black line) in axial rotation between (**A**) with blue lines and (**B**) with red lines.

The 2D angle of rotation variation after virtual reduction was measured on the XY plane using the 3D angulation set from Planes 1 2 (2′). Figure 9 shows the results of the measured angles of axial rotation and the variation. Figure 9A,B demonstrate the angles of axial rotation of the original objects (blue line) and the virtually reduced objects (red line), respectively. The overlapped results for both cases (blue line: the angle of the axial rotation measured in the original object, red line: the angle of the axial rotation measured in the virtually reduced object) are shown in Figure 9C. The rotation variation is represented by a black line in Figure 9D. The mean angle of axial rotation was 1.27° with SD: 1.19° The maximum and minimum variations were 3.80° (Case 13) and 0.00° (Case 4), respectively. There was no statistical difference (h = 0) between the two samples with a *p*-value of 0.9852 analyzed by the paired *t*-test.

## 4. Discussion

Virtual reductions using 3D object images can provide insight and guidelines for surgery strategy planning in cases of trauma. Such surgical planning is especially more helpful and effective in highly complex trauma cases [45]. Thus, to use patient-specific fixation devices created using a 3D printer, an accurate model for virtual reduction is required. For this reason, an accurate evaluation of the virtual reduction before the operation can promote more precise surgery, and quantitative assessments of the accuracy will lead to reliable surgery results. We evaluated the accuracy of 3D image-based virtual reductions according to four criteria. The criteria from the AO Surgery Reference are focused on the reduction of the tibia. However, because the accuracy evaluations in many other studies [46,47,48,49] of long bone reductions used the same criteria, we also performed the evaluations using the above criteria.

The length variation results showed that the maximum variation was below 1 mm. This variation in all cases showed the relatively high accuracy of the criterion for length. However, some cases showed length variations below 0 (negative value). This means that the length of the reduced bone was shorter than the original length. When the virtual reduction is performed in a 3D space because there is no tactile impression between the bones by the reduction, part of the object might invade the internal space of another object. Although the results of this study cannot absolutely represent the major relation between the length variation and the type of fracture, most of the results in this study showed a bigger difference of the length variation in the case of comminuted fracture than the case of single fracture. In the case of the comminuted fracture, because the virtual reduction is one of processes for the relocation of the bone fragment, there are obviously more chances to occur the length variation when the virtual reduction is progressed, due to the several bone fragments. If the human who performs the virtual reduction sensitively considers the collisions between bone fragments or the invasion of fragments to other fragments, the above trend will be more remarkable.

Originally, the concept of apposition in bone reduction was the degree of conformity of the side of the bone body between different fragments. Hence, we used the central point of projected triangles, which were composed of three marking holes. The movement of the central point corresponded to the movement of the side of the bone body. This one point could provide information on the degree of conformity of the side of the bone body. In the apposition variation results, there were only two cases over 1 mm in total apposition variation length. However, because the mean variation was less than 0.5 mm, the range of apposition variation was acceptable for the clinical application. The direction of apposition was varied and there were no specific trends for the direction. However, the trend in apposition variation was similar to the trend in rotation variation. The reason for the similar tendency was that changes in the coordinates were limited to the XY plane only.

Different criteria were applied to alignment and rotation evaluations in this study. The evaluation of alignment was divided into AP angulation and lateral angulation. Moreover, the AP angulation, lateral angulation, and rotation variation results originated from the same 3D angulation vector. We found the greatest difference in AP angulation in Case 13 (9.35°). Although the difference seemed not to be acceptable for clinical application, the difference in 3D angulation was only 0.11° (the worst case was 0.54°) because compensation from the lateral angulation was applied. Moreover, when several orthopedic surgeons who were not aware of the AP angulation saw the registered image between the original object and the virtually reduced object, they had difficulty finding the tilting point in the AP view. The reason for the difficulty was that the difference in the 3D angulation was too low to identify the tilting point. Because orthopedic surgeons cannot know the original shape of the patient’s bone when they view an X-ray image or CT image of a fractured bone, and although the ultimate shape of the reduced bone after surgery might look good, surgeons can miss the tilting point by the hidden angle in the 3D angulation. For example, although Cases 8 and 14 showed good ultimate shapes, both cases reached almost 5° AP angulation. In this case, the decision on accuracy can be evaluated differently according to the orthopedic surgeons. Although both cases had similar results in that the ultimate shape looked good, the completely different results regarding the bone union and the structural changes in the body can be caused by one factor. The results of the lateral angulation and axial rotation showed good performance when compared to the AP angulation results. The influence of the X-axis was the most impactful factor in AP angulation. Most movements of the cortical marking holes were remarkable on the X-axis after virtual reduction. The mean AP angulation variation for both and the mean angle of axial rotation were reported at almost the same level with each SD which is smaller than mean. However, the only mean lateral angulation variation showed smaller value than its SD. The reason for this result is that there are abnormally large values or small values by comparing with most of the values. In the case of the mean lateral variation, the only one value showed an abnormally large value from the data list.

Reduction evaluations have been performed using AP/lateral X-ray images to date. The results in this study are meaningful in that methodology for the evaluation of reductions in 3D coordinates was introduced. The rotation variation was especially hard to measure using the general evaluation method. However, the rotation variation after the virtual reduction in this study was presented as a quantified assessment. The alignment results showed that specific point in that the measurement of the AP/lateral angulation on the 2D X-ray image has probability for the inaccuracy of evaluation of the reduction. Moreover, 2D X-ray images include disorder factors such as the dispersion of the X-ray, grid, positioning, etc. For this reason, the accurate evaluation of alignment requires validation based on 3D angulation rather than the measurement of AP/lateral angulation on the 2D image. Nevertheless, because we cannot ask all patients to undergo CT scanning after surgery to evaluate the reduction outcome, we need to adopt a proper evaluation method according to the condition of the patient.

This study had a limitation in that the virtual reduction was manually performed and the results could change depending upon the engineer who is conducting the virtual reduction. For this reason, the numerical results in this study cannot be absolute indicators for evaluating the virtual reduction. We tried to provide a method to evaluate the accuracy of virtual reductions using quantified assessments. However, because the results showed high accuracy in the virtual reduction, it is possible that this methodology may be applied to establish reliable surgical plans. In addition, it will be helpful to fabricate patient-specific devices using a 3D printer without the mirroring technique. Because the mirroring technique needs additional CT scanning of the opposite part (or through a wide field of view) to extract the specific structure, the reliable virtual reduction results can be a good resource for fabricating devices using a 3D printer.

From all of the results, we provide several visions to use the virtual reduction at the clinical field as follows. First, the reliability of the virtual reduction using the proposed method was secured due to high-level reduction quality. The virtual reduction using the actual patient image means only relocation of the bone fragment because there is no original bone image of the patient. Then, the comparison between the original image and the reduced bone image is naturally impossible. If the proposed method can show high accuracy, the results of virtual reduction using the patient image can also show the high-level reduction quality using the proposed method with high probability. Second, the reliability of the virtual reduction using the proposed method leads to efficient operation. Because the shape of the virtually reduced bone is almost the same with the shape of the original bone, the selected fixation device will be more proper to fit to the bone than the surgical preparation using only a patient image which does not include the feature of the reduced bone, with high probability. Third, if the method of artificial intelligence (AI) based automatic virtual reduction is applied, the results in this study will be used as the index of the evaluation for this AI. The development of technology will remove manual work step by step, and the proposed method for virtual reduction in this study will also be replaced by AI. At that time, the performance of the AI can be compared with the results of this study to show the reliability.

## 5. Conclusions

In this study, the accuracy of 3D image-based virtual reductions and their usefulness were investigated through the quantitative assessments. The accuracy evaluations had four categories including length, apposition, alignment, and rotation, and we measured all four categories using six cortical marking holes on the bones. Although virtual reduction can provide insight for surgical planning before the operation, the accuracy of the reduction plan must have reliability for clinical application. The method of accuracy evaluation used in this study can be helpful in establishing a reliable plan. In the future, we will evaluate more cases to provide quantified indicators for reduction plans. 

## Data Availability

Data can be obtained from the corresponding author upon reasonable request.
